# Deep Learning for Clothing Style Recognition Using YOLOv5

**DOI:** 10.3390/mi13101678

**Published:** 2022-10-05

**Authors:** Yeong-Hwa Chang, Ya-Ying Zhang

**Affiliations:** 1 Department of Electrical Engineering, Chang Gung University, Taoyuan City 333, Taiwan; 2 Department of Electrical Engineering, Ming Chi University of Technology, New Taipei City 243, Taiwan

**Keywords:** clothing style recognition, deep learning, one-stage detection, YOLO

## Abstract

With the rapid development of artificial intelligence, much more attention has been paid to deep learning. However, as the complexity of learning algorithms increases, the needs of computation power of hardware facilities become more crucial. Instead of the focus being on computing devices like GPU computers, a lightweight learning algorithm could be the answer for this problem. Cross-domain applications of deep learning have attracted great interest amongst researchers in academia and industries. For beginners who do not have enough support with software and hardware, an open-source development environment is very helpful. In this paper, a relatively lightweight algorithm YOLOv5s is addressed, and the Google Colab is used for model training and testing. Based on the developed environment, many state-of-art learning algorithms can be studied for performance comparisons. To highlight the benefits of one-stage object detection algorithms, the recognition of clothing styles is investigated. The image samples are selected from datasets of fashion clothes and the web crawling of online stores. The image data are categorized into five groups: plaid; plain; block; horizontal; and vertical. Average precison, mean average precison, recall, F1-score, model size, and frame per second are the metrics used for performance validations. From the experimental outcomes, it shows that YOLOv5s is better than other learning algorithms in the recognition accuracy and detection speed.

## 1. Introduction

Artificial intelligence and deep learning with the research of related technologies have been rapidly growing. Image recognition has not only appeared in industry but also in daily life, such as the automatic license plate identification system in a parking garage [1], and the maturity of fruits and vegetables [2], etc. In the fashion industry, image recognition can assist in clothing design, clothing accessories collocation and related data analysis and integration [3]. Due to the rise of e-commerce, online consumption has become a popular consumption behavior with the benefits of time and cost savings. One way the clothing industry can attract consumers is the focus on visual feelings, so the style of image presentation is very important. The official website of a clothing store can show the color and style of the clothes. Furthermore, they could display many try-on pictures and a selection of accessories. This should help the customers who make the clothing matching easier. There are also many applications in agriculture, such as automatic identification of fruits [4], the quality detection of cherries [5], and the identification of the maturity of strawberries [6]. In the research of [7], pests and diseases can be identified from the inspection of the leaves of sweet peppers. Other interesting applications can be also found, such as the identification of trees infested by beetles [8], tomato virus [9], the position of fruit picking [10], and apple picking by robots [11].

Recently, one-stage objection has attracted a lot of attention, such as YOLO and SSD [12]. The emergence of YOLO has greatly improved the speed of object detection. The application areas of YOLO are very wide. For example, in the medical field, related identification applications include cervical cancer [13], blood cells [14], colorectal cancer [15], venipuncture [16], etc., Applications also include the auxiliaries for hearing and visually impaired people [17] and sign language identification [18]. Furthermore, due to the influence of epidemics, there were many mask identification studies based on the use of different algorithms [19,20,21,22]. There has been a lot of interest in self-driving cars such as automatic navigation [23], driver’s assistance [24], vehicle detection [25], vehicle tracking [26], and blind-spot detection [27]. The use of deep learning for target identification improves the accuracy of identification and also accelerates the speed of identifying targets. In the study of [28], 42 different patterns of traditional clothes were considered, and the classification accuracy was higher than 90% by using convolutional neural networks. In [29], VGG19 was used as the feature extractor to identify the Indonesia dress pattern. Faster R-CNN and some other convolutional neural networks were considered for the recognition of traditional handicraft weaving patterns. The results of the study showed that the accuracy of Faster R-CNN reached 82.14% [30].

In [31], the discrimination of clothing types were addressed, where the multilayer perceptrons and convolutional neural networks were applied on the Fashion-MNIST dataset. Moreover, images from DeepFashion and FashionMNIST datasets were selected for the recognition of clothing styles, and YOLO and ResNet were used for the study of accuracy improvement [32]. In the application of a surveillance system of clothing recognition [33], categories like suits, shirts, and jeans, etc., were considered, where the average recall rate was 80%. The study in [34] showed that with the combination of batching normalization and residual skip connections, CNNs can make the overall improvement of accuracy up to 92.54%. In [35], the R-CNN network framework combined with Softmax was applied for extracting features of shirt images, and the results indicated that an accuracy of 73.59% and a recall rate of 83.84% can be attained. In the research of clothing recognition using deep learning techniques, DeepFashion and DeepFashion2 are two datasets that have attracted lots of attention [36,37,38]. For example, fashion style recognition can help e-commerce clothing retrieval and recommendation. In order to solve the problem of classification errors caused by the same style of clothing images in different visual appearances, a joint fashion style recognition model was proposed, which was verified using the DeepFashion dataset [37]. In practice, it is necessary to establish the target object before performing garment inspection. To reduce the lengthy process of labelling, a R-CNN network was used to resolve this problem, where the DeepFashion2 dataset was considered for verification [38].

In [39], a CNN model was proposed for clothing classification, where the algorithms YOLOv3 and Tiny YOLO were analyzed. Furthermore, a learning framework was proposed for automatically classifying clothing genres based on the visually differentiable style elements [40]. In [41], an imbalanced deep learning framework was presented for large scale visual data learning, where a class rectification loss function was characterized by batch-wise incremental minority class rectification with a scalable hard mining principle. In [42], data mining and symmetry-based learning techniques were addressed to create a classification model for predicting the garment category. Based on a fashion attributes recognition network, the multi-task learning framework to improve fashion recognition was proposed to leverage the noisy labels and generate corrected labels [43]. In [44], both deep learning and image processing techniques were applied to automatically recognize and classify logos, stripes, colors, and other features of clothing. An intelligent fashion technique based on deep learning for efficient fashion product searches and recommendations were proposed, including a sketch-product fashion retrieval model and a user preference fashion recommendation model [45]. In [46], CNN networks were used to train images of different fashion styles, in which the performance was validated using the Fashion-MNIST dataset. In addition, the problem of landmark point detection in clothes was considered, where a deep learning framework was proposed to predict clothing categories and attributes [47]. In [48], CNN networks combined with a self-attention mechanism were proposed to represent clothing attributes that were more fine-grained.

The target objects of this study are the tops of clothes. The sample images are selected from the datasets of fashion clothes, DeepFashion and DeepFashion2, and Web crawling. The styles of the chosen objects are divided into five categories: plaid; plain; block; horizontal; and vertical. Google Colab virtual machine is adopted to complete the learning model training and testing. Both the two-stage and one-stage object algorithms, R-CNN and YOLO series, are discussed for performance comparisons. The differences of the R-CNN and related modified ones like Fast R-CNN and Faster R-CNN will be concisely explained. Furthermore, the key concepts and crucial differences in YOLOv1~YOLOv5 will be highlighted. The main contributions of this paper are listed as follows:

1. The process of building an integrated environment based on Google Colab is concisely explained so that those interested in deep learning can easily get involved in the study, especially for beginners who lack their own powerful GPU computer;
2. The crucial differences among R-CNN, Fast R-CNN, and Faster R-CNN are explained concisely such that readers can have easier access to the key concepts of typical two-stage algorithms;
3. The essential modifications in the development of the YOLO series are succinctly explained such that the readers will know better about the cores of each generation of one-stage YOLOs;
4. Experimental results about the recognition of clothing styles are provided along with each essential step. Furthermore, the integration of experimental outcomes are given for performance validations. The indexes of average precision (AP), mean average precision (mAP), recall, F1-score, model size, and frames per second (FPS) are investigated.

## 2. Materials and Methods

### 2.1. Object Detection

In general, object detection algorithms can be classified into different groups in accordance with one- or two-stage, whether to use anchor frames, and the labeling methods. The YOLO series and SSD are typical one-stage algorithms, while the popularly addressed two-stage ones are R-CNN, Fast R-CNN and Faster R-CNN. Anchor boxes are a set of predefined boxes that are helpful to identify the detected objects with the information scale and aspect ratio. Anchor-based algorithms include YOLOv2 to YOLOv5, Faster R-CNN and SSD, as shown in Table 1 [49]. As the labelling techniques, regional proposals are commonly adopted in R-CNN series. On the other hand, the intersection of union (IoU) is the labelling method used in YOLOv2~YOLOv5.

#### 2.1.1. Two-Stage

**Figure 1:**
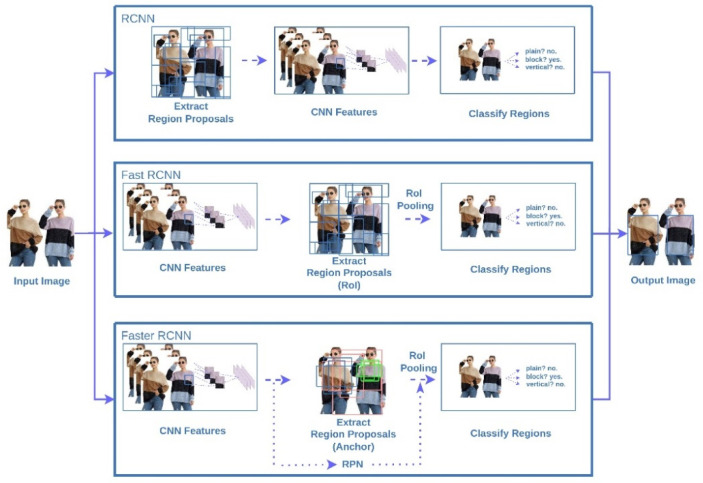
Two-stage object detection algorithms.

R-CNN, Fast R-CNN, and Faster R-CNN are typical two-stage detection algorithms, where the feature extraction and classification are two unique steps for object detection. In Figure 1, the concept of Regional of Interest (RoI) was proposed in Fast R-CNN such that the feature extraction can be more efficient. To further reduce the detection time, a Regional of Proposal Network was presented for Faster R-CNN. Due to the increase in architecture complexity, Faster R-CNN requires high efficiency computation capability for real-time object detection.

#### 2.1.2. One-Stage

**Figure 2:**
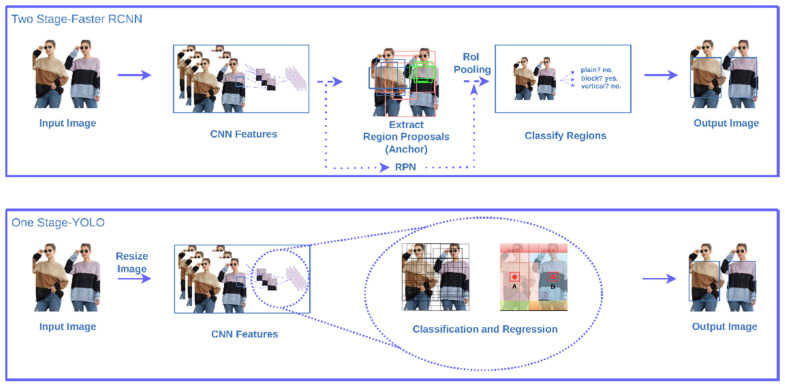
Comparison between one-stage and two-stage object detection algorithms.

The You Only Look Once (YOLO) series, first presented in 2016, are one-stage objection algorithms. Compared to the two-stage Faster R-CNN, the regression-based classification is used to replace the RoI pooling layer such that detection time can be reduced, as shown in Figure 2. YOLOv5 is a newly developed algorithm in the YOLO series. Basically, YOLOv5 has a relatively small size that will make the implementation on mobile devices more feasible.

YOLO is a family of object detection architectures and models pretrained on the COCO dataset. In contrast to the two-stage detectors based on the region proposal method, the representative one-stage detector, YOLO, uses the idea of regression to predict all the categories along with the corresponding confidence and bounding box information, which can speed up the detection greatly, although this comes at the expense of slightly reduced precision. YOLOv5 is a state-of-art deep learning framework, and the whole network is composed of four parts: input; backbone; neck; and head. In the series of YOLOv5, YOLOv5s has the benefit of less model size that would provide the potential for future interesting applications such as edge AI and machine learning on a micro-controller unit (MCU). In this paper, the details about the key technologies and how to build the YOLOv5s machine learning framework will be discussed. Furthermore, the feasibility will be validated for the clothing style recognition. The performance including the computation cost and recognition accuracy will be compared with the YOLOv3, YOLOv4 and traditional two-stage R-CNN frameworks.

### 2.2. Implementation of Deep Learning Framework

#### System Built with Google Colab

**Figure 3:**
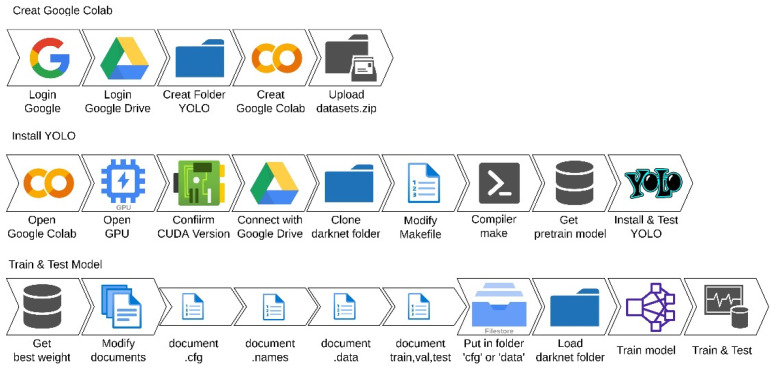
Implementation of YOLOv1~YOLOv4 for model training and testing with Google Colab.

Deep learning usually relies on a GPU computer for high-efficiency computation. For beginners who are interested in the deep learning topics, system building may not be affordable. In this paper, an open-resource Google Colab is adopted to build the environment for learning model training and testing. In Figure 3, three steps are performed for the YOLOv1~YOLOv4 installation and corresponding model training and testing. In the creation of the Google Colab project, a dataset is required to be well-prepared and uploaded into the created YOLO folder. Next, the user must confirm all the settings of the GPU and CUDA before the installation of YOLO algorithms. Finally, provided with the pre-defined weights, the model training and testing of the selected YOLO algorithm can be sequentially performed. It is noticed that Darknet is the neural network framework here.

**Figure 4:**
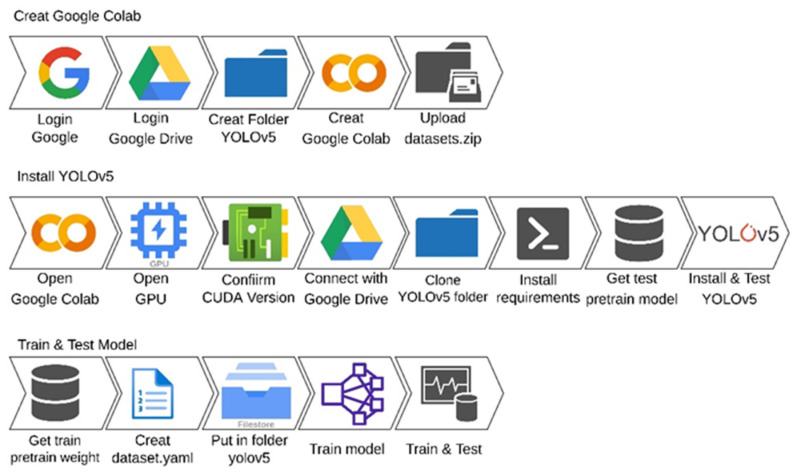
Implementation of YOLOv5 for model training and testing with Google Colab.

The building process of YOLOv5 is like the process in Figure 3. While installing the environment of YOLOv5, the neural network framework is PyTorch, as shown as Figure 4. A typical YOLOv5 series includes YOLOv5x, YOLOv5l, YOLOv5m, and YOLOv5s. In the comparison between each other, YOLOv5s has a minimum model size.

### 2.3. YOLO Algorithms

You Only Look Once (YOLO) is a typical one-stage object detection algorithm. It is formulated as a regression problem, from which the bounding boxes and class probabilities can be predicted directly from full images in one evaluation [50]. The first generation of YOLO, YOLOv1, was proposed in 2016 by Joseph Redmon [51]. Compared to the two-stage object detection methods, YOLO series have significant improvements in detection speed and model size. In YOLO series, Darknet is the deep learning framework adopted for YOLO v1~v4, while the framework of YOLOv5 is PyTorch. YOLOv1, inherited from GooLeNet, considers the objection detection as a regression problem. The processing speed is fast, but the recognition accuracy is less than the two-stage algorithms. In particular, the recognition outcomes of small objects need a certain degree of improvement. Based on YOLOv1, YOLOv2 has shown some progress on accuracy and processing speed with the use of Darknet-19 framework. However, the recognition of small objects is still unsatisfactory. In YOLOv3, a Neck module is added for feature fusion, the output dimension is increased to three, and the recognition of small objects is better. YOLOv4, combined with many technical studies and experimental results, provides better accuracy of object detection. YOLOv5 is a newly developed algorithm, which has a much smaller model size than the aforementioned algorithms [52].

**Figure 5:**
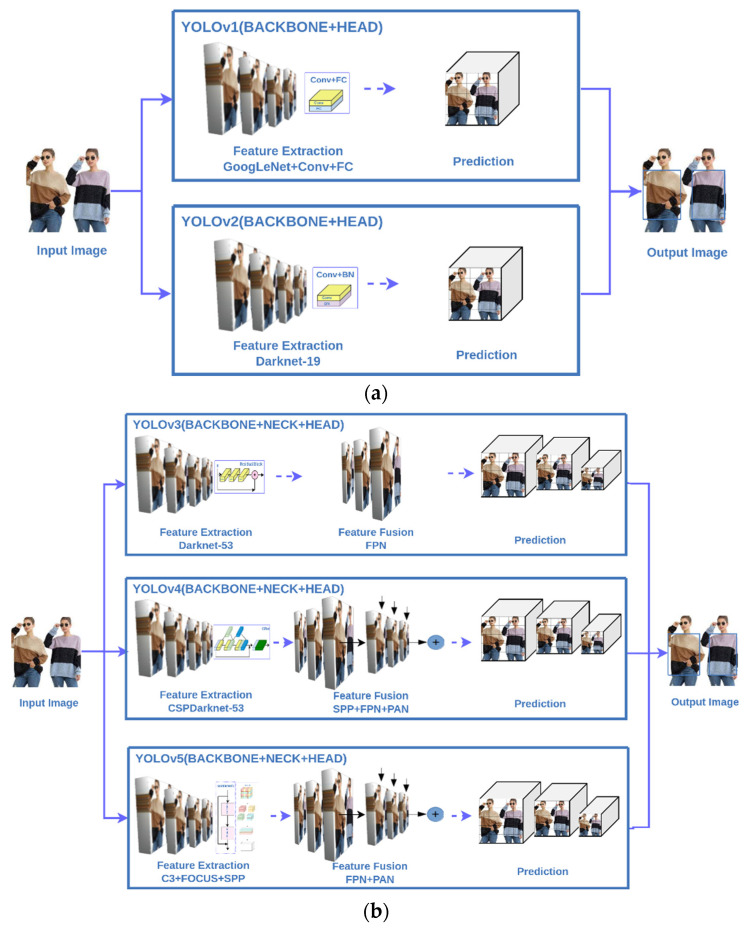
Architecture of YOLO: (**a**) YOLOv1~YOLOv2, (**b**) YOLOv3~YOLOv5.

Backbone, Neck and Head are the main modules in YOLO. To concisely explain the key differences in the YOLO series, the scheme diagrams of YOLOs are shown in Figure 5. In Figure 5a, it can be seen that YOLOv1 basically uses the traditional convolutional layer and a fully connected network to extract features. On the other hand, the feature extraction of YOLOv2 is performed with a Darknet network. In YOLOv3~YOLOv5, a certain feature fusion is added that can integrate related information extracted from a group of images without losing data. The feature extraction and fusion methods of YOLOv3~ YOLOv5 are shown in Figure 5b [53,54,55]. The function of FOCUS is mainly to increase the speed, where the image will be sliced and rearranged. SPP, known as the spatial pyramid pooling method, solves the problem of picture distortion caused by image deformation stretching or cropping, that greatly improves the speed of generating candidate boxes and the computational cost reduction. In addition, the use of FPN (Feature Pyramid Network) and PAN (Path Aggregation Network) will complete the feature fusion of high and low layers, so that object detection can be improved, especially in the detection of small objects.

## 3. Results

### 3.1. Integrated Develepmental Environment

**Figure 6:**
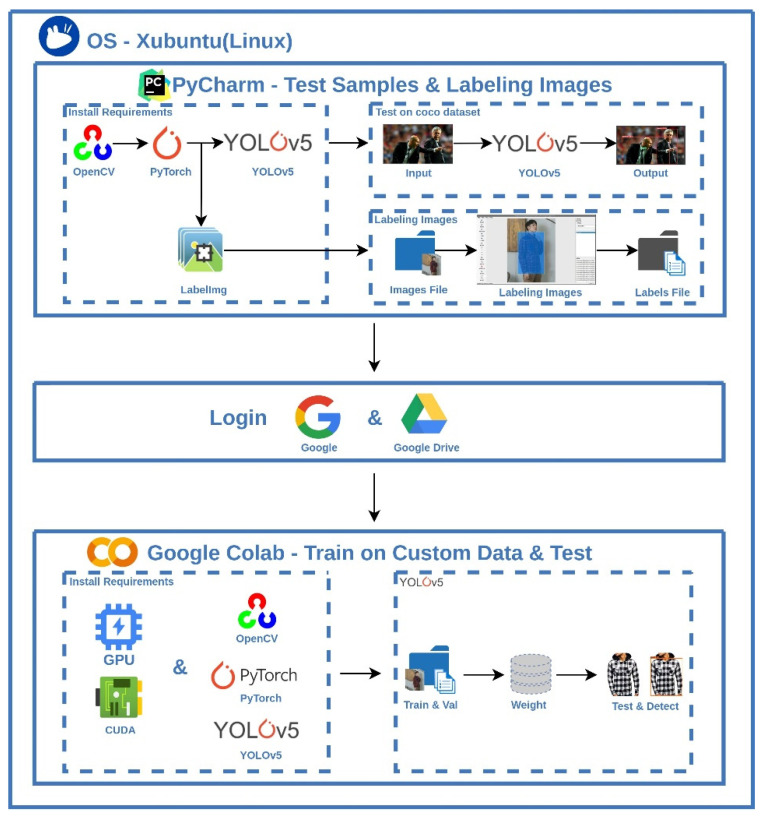
The scheme of the proposed YOLOv5s deep learning environment.

**Figure 7:**
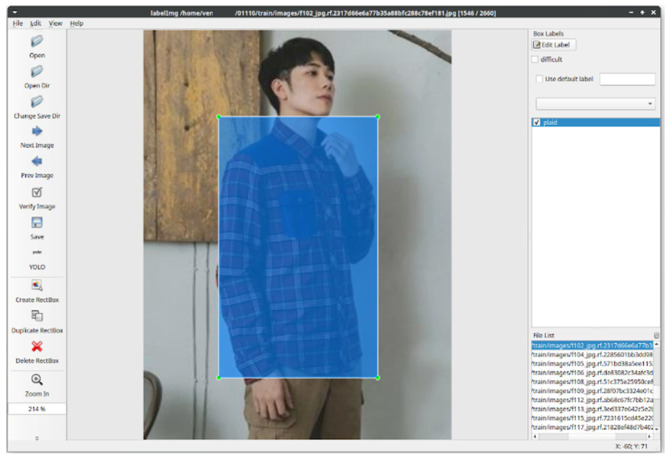
Outcome of image labelling.

The whole scheme of developed environment for YOLOv5s is shown in Figure 6. Google Colab is used to build the experimental environment, where the operating system is Xubuntu [56]. Xubuntu is an elegant and easy to use operating system, coming from Xfce, which is a stable, light and configurable desktop environment. In addition, the virtual GPU is Nvidia Tesla K80 with CUDA 11.2 and OpenCV 4.1.2. PyCharm is a Python based development environment providing many essential tools for Python developers, tightly integrated to create a convenient environment to access the command line, connect to a database, create a virtual environment, and manage your projects [57]. For the image labelling, LabelImg is a graphical image annotation tool, written in Python and using PyQt for its graphical interface. Annotations are saved as XML files in PASCAL VOC format. Moreover, it also supports YOLO and CreateML formats [58,59]. During the labelling process, the area of object and its class belonging will be determined. The information format is selected to fit the YOLO format. This information contains the class, x-y coordinate, and length-width of objects, as shown in Figure 7.

### 3.2. Dataset

**Figure 8:**
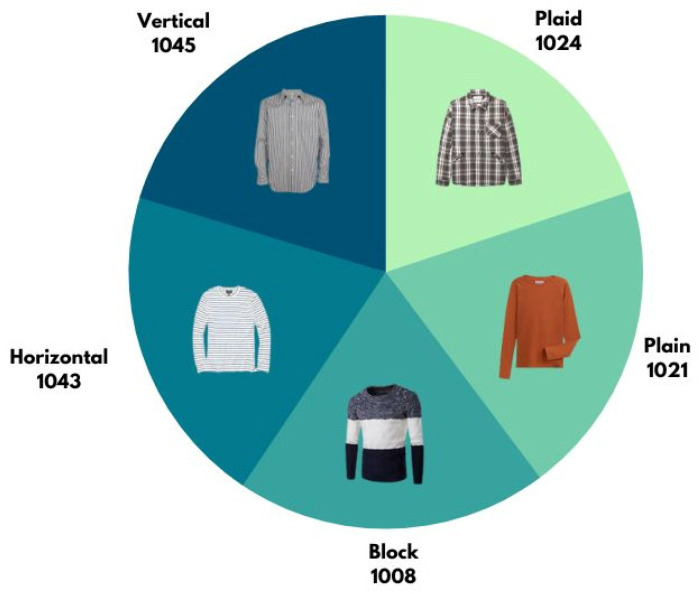
Image samples of each category of shirts.

In this paper, the image datasets are collected from five open resources. For example, DeepFashion is a dataset of 50 classes fashion clothes, where the total number of images is over 80 million [60]. In addition, DeepFashion2 contains 49.1 million images of 13 classes of clothes [61]. Moreover, image samples are also collected from Google pictures and the sites of web fashion shops. The initial amount of clothing samples is 4455. With additional data augmentation, the number of total image samples used for this research is 5141, shown in Figure 8. The image samples are categorized into five groups: plaid; plain; block; horizontal; and vertical. In Figure 8, the number of each category is indicated, such as plaid having 1024 samples, etc.

**Figure 9:**
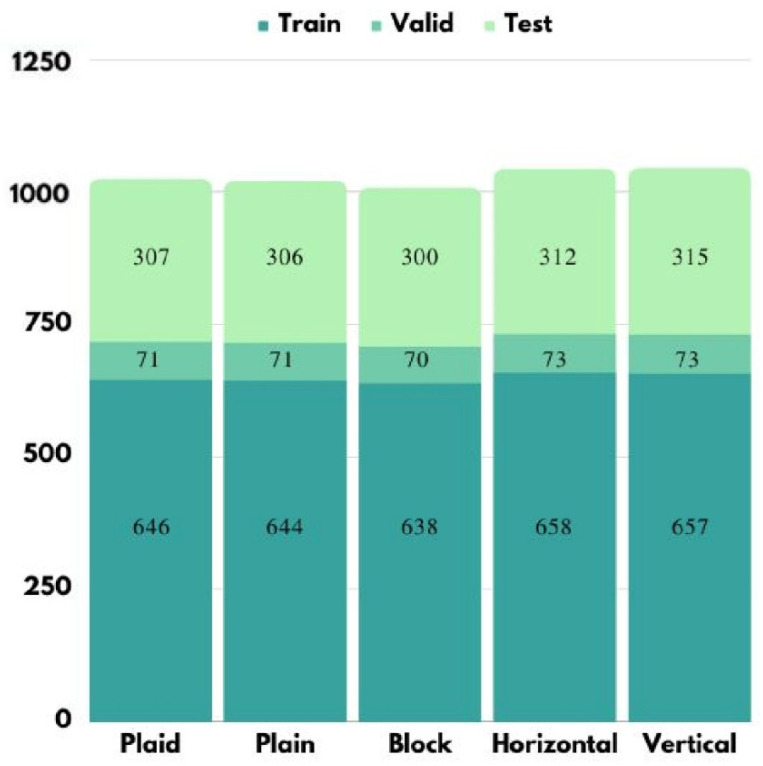
Image samples of each category in the process of training, validation, and testing.

The collected image samples will be divided into training set, validation set, and test set. Training set is used to train the learning model. Validation set is used to adjust and select models. Finally, the model evaluation is performed with the test set. According to the 60:10:30 ratio, the number of samples of each category in the stage of training, validation, and testing are shown in Figure 9.

### 3.3. Integration Testing Results

The YOLOv5 is mainly composed with four modules, i.e., Mosaic augmentation, Backbone, Neck, and Head. The corresponding experimental outcomes are illustrated in the following.

**Figure 10:**
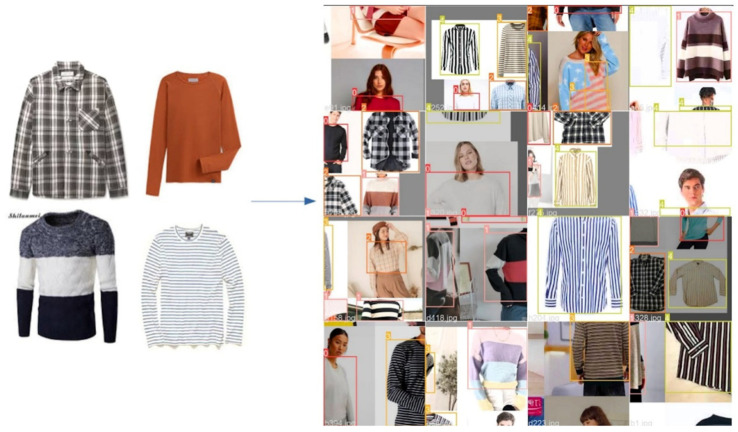
Mosaic image augmentation.

Image augmentation creates new training examples out of existing training data. It is not easy to truly capture an image for every real-world scenario. Thus, adjusting existing training data to generalize to other situations allows the model to learn from a wider array of situations. The idea behind Mosaic is simply taking four images and randomly combining them into a single image, as shown in Figure 10.

**Figure 11:**
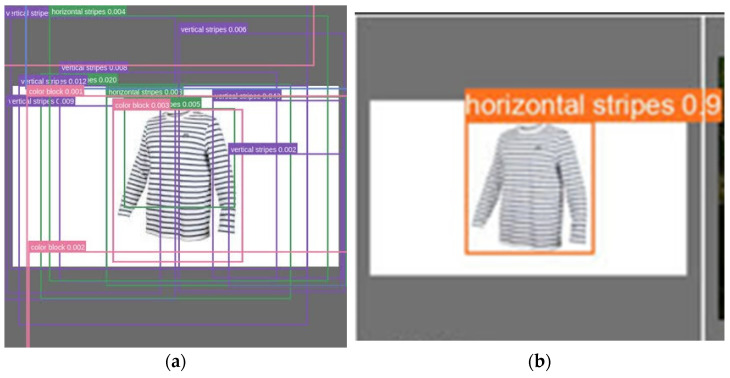
Anchor boxes: (**a**) candidates during the processing, (**b**) final choice.

In object detection, bounding boxes are usually used to describe the spatial location of an object. For solving the problem of objects overlapping in an image, YOLO uses different sizes of rectangles to the anchor boxes. Basically, for each anchor box, one can calculate which object’s bounding box has the highest overlap divided by non-overlap. During the training process, the predicted bounding box is iteratively compared to the ground-truth to generate the optimal bounding rectangle of an object, shown as Figure 11. In Figure 11a, each possible class is represented with rectangles of the same color. The optimal bounding box of the object is shown in Figure 11b.

**Figure 12:**
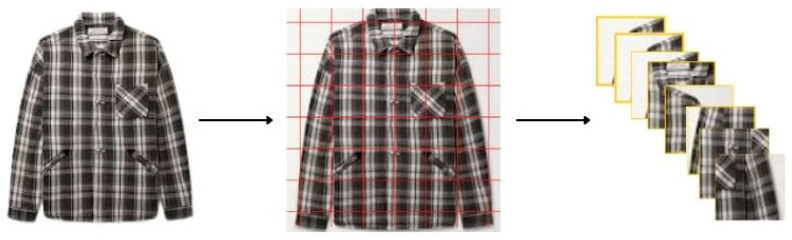
Feature extraction and fusion by Backbone and Neck.

**Figure 13:**
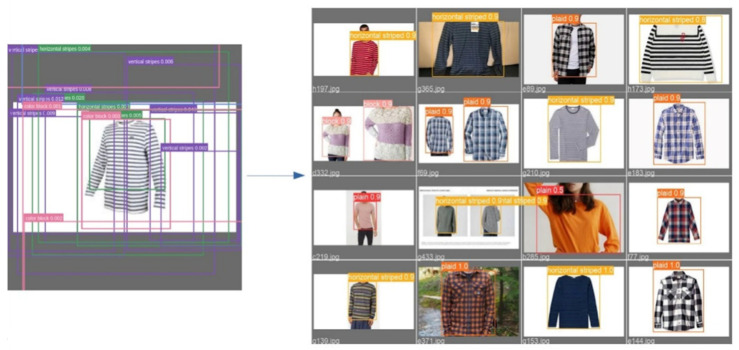
Object prediction by Head.

**Figure 14:**
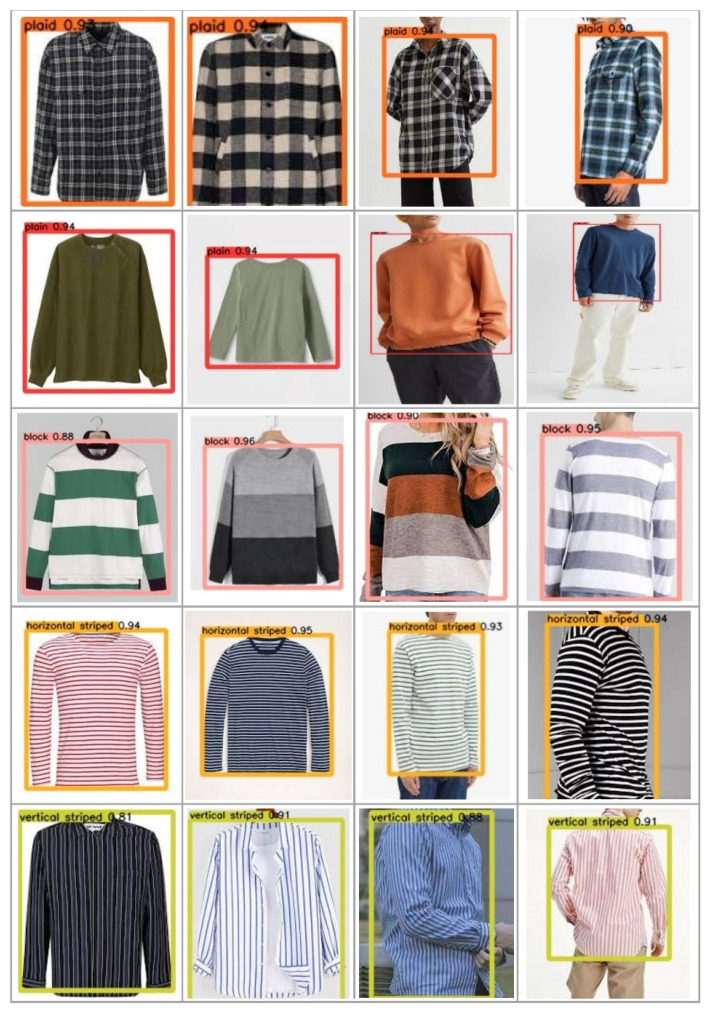
Testing results (top to bottom: plaid, plain, block, horizontal, vertical).

**Figure 15:**
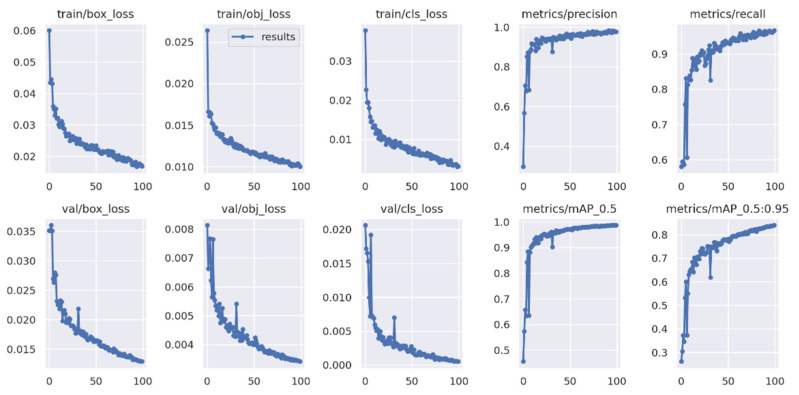
Integration results of YOLOv5s (100 epochs).

**Figure 16:**
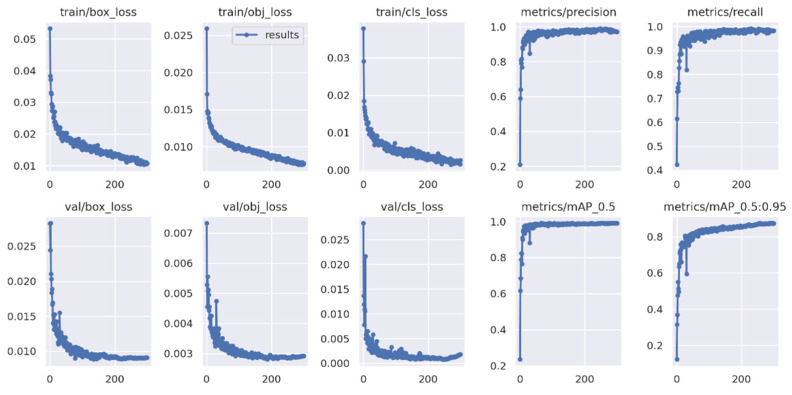
Integration results of YOLOv5s (300 epochs).

The YOLO Backbone is a convolutional neural network that pools image pixels to form features at different granularities. The Backbone in the deep learning architecture basically acts as a feature extractor. The Neck is a subset of the bag of specials, and it basically collects feature maps from different stages of the backbone. In simple terms, it is a feature aggregator. This is the part of the network that makes the bounding box and class prediction. The Head is also known as the object detector. Basically, the Head can be used to find the region where the object might be present, but with no information about which object it is. The corresponding outcomes are shown in Figure 12 and Figure 13. Furthermore, some testing results for the trained YOLOv5s model are shown in Figure 14. To validate the feasibility of the constructed YOLO learning architecture, some two- and one-stage learning algorithms are adopted for performance comparisons, as shown in Table 2 and Table 3. In Table 2, average precision (AP), mean average precision (mAP), model size, and frame per second (FPS) are addressed. The metrics of precision, recall, and F1-score are shown in Table 3. In the training and testing processes, the losses and performance metrics of each epoch are shown in Figure 15, where the number of epochs is 100. From the outcomes in Table 2 and Table 3, the YOLOv5s has better performance in all metrics of interest. Furthermore, the metrics in the training and testing processes of YOLOv5s with 300 epochs are shown in Figure 16. In Table 2 and Table 3, both the metrics for performance validations of YOLOv5s with 100 epochs and 300 epochs are included. Efficiency is generally improved with more epochs; however, the model size is slightly increased.

## 4. Discussion

In this paper, one of the main concerns is to concisely demonstrate the essential steps to build a model training and testing environment on Google Colab. The authors sincerely hope that the guidance can help readers without the need for expensive computation supports. Based on the authors’ experience, the following comments could be helpful. For instance, while getting access to Google Colab, the selected GPU in the virtual machine must be enabled. Your own Google Drive is connected, and the file paths are correct. Furthermore, the version of each adopted software program like the operating system and framework must be compatible with each other. In the stage of building the YOLO dataset in the cloud environment, the self-defined storage path needs to be identified. The labelling outputs must meet the YOLO format. In practice, the filename of images (.jpg) and the labels (.txt) must be consistent. Furthermore, the user needs to install application programs according to the content shown in requirements.txt. To avoid careless mistakes, it is suggested that a small number of epochs is first chosen. After the correctness of all settings has been confirmed, a bigger number of epochs can be then used in the process of model training.

In practice, during the validation process, a better model with related parameter settings can be obtained. Then the selected model will be used for performance testing. Using the three-category dataset division, train–validation–test, the problem of overfitting can be avoided. Furthermore, the generalized errors of the corresponding machine learning can be reduced. In real-time applications, YOLO can also be workable with embedded devices, such as Raspberry Pi, Nvidia Jetson TX2, and Arduino UNO [62,63,64,65,66]. The implementation of state-of-art lightweight algorithms, like the YOLOv5s addressed in this paper, is an emerging topic in deep learning. In this paper, a complete and easy to use open-source environment has been built. In the future, it can afford the needs of enhanced applications, such as the addition of color recognition into the recognition of clothing styles. Furthermore, the generative adversarial network (GAN) can be considered to improve the capability of small-object detection of YOLOs.

In summary, the essential concepts of the object detection algorithms are highlighted in the following. The key steps of the object detection mainly include the area identification and category classification. Two-stage object detection algorithms consider these steps as two unique execution processes, while the one-stage algorithms merge these two steps into one process. In general, a high recognition accuracy of two-stage detection algorithms can be obtained, but the whole process is generally time-consuming. In the one-stage algorithms, a regression-based classification is used such that the detection time can be reduced without losing detection accuracy much. Due to the high complexity of two-stage algorithms, the computing cost of the relevant model training increases as well. R-CNN, Fast R-CNN, and Faster R-CNN are typical two-stage object detection algorithms. In R-CNN, selective search algorithm is used to locate the candidate regions in images for the consequent feature extraction. Basically, this process is very time-consuming as a result of repeated feature extraction in overlapping areas. Since each candidate region will be processed by the convolutional neural network, the computation burden is increased along with the amount of increasing candidate regions. In Fast R-CNN, only one iteration of the CNN computation is required for the feature extraction of input images so that the computation cost is reduced. In Faster R-CNN, to further improve the computation efficiency, a RPN network is considered to replace the selective search method, thus the detection accuracy and speed is increased.

In the YOLO series, the input images are resized into a format of 448 × 448, and then sliced into several 7 × 7 grids. Following the CNN feature extracting, non-maximum suppression (NMS) is used to filter out less confident bounding boxes. In YOLOv1, input images are first sliced into grid cells. In each grid cell, only two bounding boxes are used for class prediction. Through the subsampling process, the image in the last layer is much smaller and makes object recognition become difficult. Alternatively, anchor boxes are used for the prediction of bounding boxes in YOLOv2. To improve the recognition capability for small objects, a passthrough layer is added in the second to last CNN layer. In YOLOv3, a Neck module is adopted for multi-scale feature fusion. Thus, the recognition efficiency of small objects is significantly improved. In YOLOv4, a cross stage partial network (CSPNet) is used for feature extraction. In addition, the use of FPN and PAN will complete the feature fusion, so that the efficiency of object detection can be improved, especially in the detection of small objects. Finally, in YOLOv5, the addition function of FOCUS is mainly to increase the detection speed, and the SPP greatly improves the speed of generating candidate boxes and the computational cost reduction.

## 5. Conclusions

In this paper, a lightweight learning algorithm, YOLOv5s, is considered for the recognition of clothing styles. YOLOv5s is a one-stage objection method that the superiority of detection speed and model size is the main concern. An open-source integration development environment on Google Colab is built for model training, validation, and testing. The image samples are collected from either datasets of fashion clothes or Web searching of online clothing shops. The integrated process about how to build a free computing environment is concisely explained. The readers who are interested in deep learning may be easily able to build their own environments in various applications. Experimental results illustrate that the one-stage object detection algorithm YOLOc5s has the benefits in many metrics, such as mAP, precision, recall, F1-score, model size, and FPS.

## Figures and Tables

**Table 1:** Object detection algorithms [49].

Stage	One-stage	YOLOv1~YOLOv5, SSD
	Two-stage	R-CNN, Fast R-CNN, Faster R-CNN
Anchor	Anchor-free	YOLOv1
	Anchor-based	YOLOv2~YOLOv5, SSD, Faster R-CNN
Labelling	Regional proposal	R-CNN, Fast R-CNN, Faster R-CNN
	Key point	YOLOv1
	IoU	YOLOv2~YOLOv5, SSD, Faster R-CNN

**Table 2:** Performance comparisons of different deep learning algorithms.

	AP (%)					mAP (%)	Model Size	FPS
	Plaid	Plain	Block	Horizon	Vertical			
Faster R-CNN	87.0	93.3	100	85.9	85.7	90.3	175.5	7
YOLOv3-tiny	89.7	95.7	90.0	92.0	91.3	91.7	33.4	28.5
YOLOv4-tiny	98.9	98.0	94.1	93.0	97.0	96.2	23.1	24.5
YOLOv5s (100 epochs)	99.3	99.1	99.0	97.3	94.9	98.4	14	40
YOLOv5s (300 epochs)	98.5	99.4	99.3	99.4	96.6	99.1	14.4	40

**Table 3:** Performance comparisons of different deep learning algorithms (cont’d).

	Precision	Recall	F1-Score
Faster R-CNN	0.91	0.89	0.90
YOLOv3-tiny	0.93	0.85	0.89
YOLOv4-tiny	0.90	0.95	0.93
YOLOv5s (100 epochs)	0.98	0.96	0.97
YOLOv5s (300 epochs)	0.97	0.98	0.97

## Data Availability

Not applicable.
